# Comparison of wastewater treatment plants based on the emissions of microbiological contaminants

**DOI:** 10.1007/s10661-018-7035-2

**Published:** 2018-10-18

**Authors:** Michał Michałkiewicz

**Affiliations:** Institute of Environmental Engineering, Poznan University of Technology, Berdychowo 4, 61-138 Poznan, Poland

**Keywords:** Bioaerosols, Air contamination, Wastewater treatment plants, Emission factor, Statistical analysis

## Abstract

This article presents the current and important results of bioaerosol studies which allow for the comparison of microbial contamination of air in 11 wastewater treatment plants (WWTPs), which differed in terms of capacity from 350 to 200,000 m^3^/day. The abundance of mesophilic bacteria, M+ and M− *Staphylococcus*, *Pseudomonas fluorescens*, *Actinobacteria*, coliform and psychrophilic bacteria, and microscopic fungi was determined. Additionally, the air temperature, relative humidity, wind velocity, and direction were also analyzed at each research station. The obtained very numerous results of bioaerosol and climate parameter studies were subjected to statistical analysis. The results regarding the minimum, maximum, and median abundance of the studied bacteria and microscopic fungi at 11 WWTPs and in background studies at control stations were presented in tables. Additionally, basic descriptive statistics for all studied microorganisms at specific seasons were presented. It was established that at the areas of WWTPs, the microscopic fungi were present that the highest concentrations (ranging from 0 to 1,148,530 CFU m^−3^), followed by psychrophilic bacteria (ranging from 40 to 225,000 CFU m^−3^) and mesophilic bacteria (ranging from 0 to 195,000 CFU m^−3^). The novel elaboration of bioaerosol study results based on cluster analysis and determination of a dendrogram allowed to compare the studied WWTPs. The similarity was decided based on the type of studied microorganisms and their dominance and abundance, while no similarities were observed in terms of capacity. In order to investigate the relation between the abundance of bacterial groups as well as microscopic fungi and microclimatic parameters (air temperature and humidity), a calculation of Spearman’s range correlation coefficients was conducted.

**Electronic supplementary material:**

The online version of this article (10.1007/s10661-018-7035-2) contains supplementary material, which is available to authorized users.

## Introduction

The problems associated with the contamination of atmospheric air are becoming increasingly important in the modern world. Among several scientific fields, aerobiology has been distinguished as an interdisciplinary science dealing with the study of bioaerosols, microorganisms, and biological materials associated with atmospheric air. Biological aerosols, often called bioaerosols, are colloidal systems in which the dispersed phase consists of microorganisms (bacteria, fungi, and viruses), their metabolites, toxins, and fragments which occur in the form of fine droplets or solid particles suspended in air, which serves as the dispersive phase (Bauer et al. 2002; Fuzzi et al. 2006; Tomasi and Lupi 2017). The particle diameters of bioaerosols commonly range from approx. several nanometers to approx. 100 μm, with the size of virus particles serving as the lower limit and structures of pollen or seeds being the upper limit. The problem of bioaerosols concerns several scientific fields, beginning with medicine, through botany, zoology, environmental engineering, and ecology and ending with typical microbiology (Roodbari et al. 2013; O’Connor et al. 2015). The studies conducted in Poland and throughout the world are focused on, e.g., the mechanisms of bioaerosol formation (Mandal and Brandl 2011; Tomasi and Lupi 2017; Blanchard and Syzdek 1970), bioaerosol content (Fuzzi et al. 2006; Tomasi and Lupi 2017), methods of sampling air for studies (Pastuszka et al. 2013; Gregová et al. 2008), and abundance and type of microorganisms in the indoor and outdoor environment as well as the influence of different objects on the air contamination level (Mentese and Tasdibi 2016; Dungan 2012; Wéry 2014; Górny and Dutkiewicz 2002). Bioaerosols may become a serious risk to the health of the population, mainly because airborne bacteria and fungi can cause infectious diseases as well as allergic and toxic effects. It was simultaneously established that there is a fundamental difference in the quality and quantity of microorganisms in the external and internal environment (Brągoszewska et al. 2018).

An important role in the emission of bioaerosols is played, among other, by municipal facilities including wastewater treatment plants (WWTPs). The range of influence of a WWTP on its surroundings may differ notably and the composition of wastewater, the technology used for treatment, plant capacity, and method of securing each treatment step from the spread of microorganisms to the surrounding area play a crucial role in the potential emission of bioaerosol to atmospheric air (Sánchez-Monedero et al. 2008; Korzeniewska et al. 2009; Li et al. 2012). Municipal wastewater which enters the WWTP contains numerous pathogenic microorganisms which may form bioaerosols and be lifted into atmospheric air as a result of flow, turbulence, and aeration of wastewater (Carducci et al. 2000; Thorn et al. 2002; Maki et al. 2017). Moreover, side-products such as screenings, sand, or sewage sludge formed during subsequent stages of treatment also contain hazardous, often pathogenic microorganisms which may have a negative impact on the environment, WWTP employees, and the inhabitants of local areas (Uhrbrand et al. 2011; Pringle 2013; Roodbari et al. 2013).

In classical methods of biological treatment of wastewater using activated sludge, there is no possibility to eliminate numerous chemical compounds from the wastewater, including some pharmaceuticals which may be a potential hazard for surface, ground, and drinking water. Due to this reason, it is necessary to introduce additional methods for removing contaminants, such as coagulation, membrane filtration, adsorption, or advanced oxidation processes. The literature indicates that wastewater treatment using these processes allows for satisfactory removal of many pharmaceuticals from wastewater; however, the generation of sludge and the necessity to regenerate the adsorbent are major flaws of using such processes (Ahmadzadeh and Dolatabadi 2018a). Industrial, agricultural, and hospital wastewater include numerous, difficult to remove substances, the neutralization of which requires the most novel, effective, and efficient techniques, which are friendly for the environment. The literature provides examples of new methods used for the removal of different contaminants from the wastewater, including pharmaceuticals and germicides, using processes such as electrochemical treatment with the use of the electro-Fenton (EF) process, electrocoagulation (EC) with aluminum electrodes, electrocoagulation with iron electrodes, flocculation and adsorption, or electrochemical peroxidation (ECP), which is an effective combination of the advanced oxidation process and Fenton (Ahmadzadeh and Dolatabadi 2018b; Ahmadzadeh et al. 2017a; Yoosefian et al. 2017; Ahmadzadeh and Dolatabadi 2018c; Ahmadzadeh et al. 2017b).

Aside from contaminant removal methods, the methods of determination of some wastewater parameters using electrochemical and optical methods are also used, e.g., determination of the pH value, conductivity, redox potential, wastewater temperature, oxygen concentration, nitrate compounds, turbidity, heavy metal ions content, pharmaceuticals, and biological compounds. Indicators, probes, and electrodes are used for measurements. An example of novel measurement methods includes, e.g., modification of carbon paste electrode with NiO nanoparticles and an ionic liquid and the use of ion selective electrodes with novel membranes. The used electrochemical sensors are effective and offer advantages such as low cost, rapid analysis, high sensitivity, and selectivity compared to instrumental analysis (Fouladgar and Ahmadzadeh 2016; Pardakhty et al. 2016; Soltani et al. 2016; Kassim et al. 2011).

The aim of this study was to compare 11 wastewater treatment plants based on the current bioaerosol study results, to indicate the variations in the emissions of bacteria and microscopic fungi and to determine the group of microorganisms which causes the highest contamination of air. The abundance of bacteria and microscopic fungi recommended by Polish Standards was analyzed at stations located at different treatment stages in 11 WWTPs as well as at a control station, the so-called background study, which was always located on the windward side of the studied WWTP (PN-89/Z-04111/02 1989; PN-89/Z-04111/03 1989). Such selection of the control station allowed to compare the microbial emission levels from the WWTP area in relation to uncontaminated air, which was not under the influence of the communal object.

The results of bioaerosol studies are important and novel; since they were obtained from the area of 11 different WWTPs, they were subjected to a detailed statistical analysis and they were conducted in an annual cycle during all seasons, taking the variability of climate parameters into account. All the studies were conducted in accordance with the guidelines of Polish Standards and the obtained results were used to compare of municipal objects and indicate correlations between the emissions of microorganisms and the microclimatic parameters of air.

## Materials and methods

### Characterization of the studied WWTPs

Eleven WWTPs were selected for studies of the microbial contamination of air, which differed in terms of capacity from 350 to 200,000 m^3^/day. Ten objects were mechanical-biological WWTPs with enhanced phosphorous removal, whereas one was a hydrobotanic Lemna-type treatment plant (no. 6). Additionally, a sludge composting plant was present on the area of two WWTPs (no. 1 and 11). Table 1 presents the number, type, and capacity of the studied WWTPs as well as the population equivalent (PE) and the number of research stations along with background research.

**Table 1:** Number, type, and capacity of studied wastewater treatment plants, population equivalent (PE), and number of research stations

WWTP no.	WWTP type	Capacity (m^3^/day)	Population equivalent (PE)	Number of research stations
1	MB+KO	9360	140,000	5
2	MB	9602–16,000	126,500	16
3	MB	2300	18,200	8
4	MB	5883	35,700	8
5	MB	1138	12,300	6
6	L	350	6200	7
7	MB	500	4150	8
8	MB	200,000	1,000,000	7
9	MB	5600–8000	33,000	11
10	MB	50,000 – 80,000	63,300	9
11	MB+KO	12,438 – 17,307	51,800	10

*MB*, mechanical-biological; *KO*, composting plant; *L*, Lemna-type

**Fig. 1:**
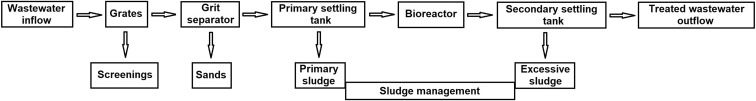
Diagram of the technological wastewater treatment system on the analyzed wastewater treatment plants

The majority of WWTPs were localized outside the direct urban area; nevertheless, some of them bordered with single residential buildings. Such localization may have caused a different influence of bioaerosol emission on the surrounding areas. The technological system of the majority of WWTPs was very similar, as presented in Fig. 1.

Additionally, a storage point for supplied wastewater was present on the area of the majority of WWTPs or in their direct vicinity. Raw wastewater flowing into the WWTP was directed to the grate building, which mostly contained mechanical, self-clearing grates with a 6-mm clearance and emergency grates with a clearance of 7 to 40 mm. The screenings detained on the grates were contained in containers and sprinkled with chlorinated lime for hygienization. Next, the wastewater entered the grit separator and the solids (sands) were transferred to a free-standing storage container. The wastewater from the grit separator entered the primary settling tanks in which the sedimentation and flotation suspensions were removed. The separated primary sludge was directed to the sludge management stage. Next, the mechanically treated wastewater entered the biological treatment area of the WWTP.

Biological treatment of wastewater was mostly carried out in bioreactors with a differing construction, which contained chambers used for, e.g., dephosphatation, denitrification, and nitrification. A single WWTP (no. 6) contained a Lemna-type aerated pond. In the majority of WWTPs, a fine bubble aeration system was used in the nitrification chambers, whereas in a single WWTP (no. 1), old Silesia 270 APC-type surface aerations were used. After the biological treatment, the wastewater was directed into secondary settling tanks in which the treated wastewater was separated from the sludge, which was recirculated into the bioreactor and in the case of a single WWPT (no. 6), a Lemna-type aerated pond was used. The excessive sludge was directed to sludge management stages. The treated wastewater entered the receiving point, which in most cases was a river or a channel flowing into the river. Within each WWTP, a more or less developed sludge management was conducted. Primary sludge and the mechanically concentrated excessive sludge were pumped into open digester tanks (ODT) or closed digester tanks (CDT), where they were subjected to fermentation. The sludge after settling was subjected to dehydration and hygienization using quicklime. Next, it was stored on a sludge storing site (no. 2, 3, 4, 7, 9), pounds (no. 1, 3, 5, 8, 10), or lagoons (no. 1, 6) and subjected to a thermal drying process (no. 8, 10) or composting (no. 1, 11). In most cases, it was periodically exported or used for the reclamation of the WWTP area (no. 2, 4, 5) and served as an organic fertilized for the production of energy willow (no. 9). On the area of two WWTPs (no. 1 and 11), there was an additional composting site for sludge and waste.

### Research method

Microbial assessment of air was conducted on the area of 11 WWPTs during an annual cycle in each season. Several air sampling stations (from 5 to 16 in Table 1) were established on the area of each WWTP, which were located on all wastewater treatment stages. These stations were located near objects (devices) which could potentially be a source of bioaerosol emissions. In most cases, the stations were located in the outdoor environment; however, they were also inside some closed objects (e.g., the hermetic grate building, enclosed bioreactor, sludge concentration building). In some cases, the air from the hermetic objects was directed to biofilters (e.g., WWTP no. 2, 8, 10). In most cases, the emission near the research stations could occur due to intense flow, transfer, aeration, or turbulence of wastewater as well as treatment of sludge and storage of screenings and grit. Precise identification of bioaerosol sources is the basis for conducting appropriate actions focused on the elimination of the causes of their formation (e.g., reducing the flow intensity and turbulence, change of the aeration system, partial or complete hermetization). During the studies, at least a single control station (so-called background research) was designated, which was located at a distance of 150–200 m on the windward side relative to the WWTP border. This allowed to confirm that the WWTP is the source of bioaerosol emissions. Microbial contamination of air was analyzed in accordance with Polish Standards: PN-89/Z-04111/02 and PN-89/Z-04111/03. Since the above-mentioned standards were invalidated in 2015 and were not replaced by new standards, the assessment of air purity may be still conducted according to these Standards. Based on the guidelines in these Standards, the following microorganisms were studied: mesophilic bacteria, hemolytic mannitol-positive (M+) and mannitol-negative (M−) staphylococci (*Staphylococcus*), *Pseudomonas fluorescens*, actinomycetes (*Actinobacteria*), and microscopic fungi. Due to the fact that the studies were carried out on the area of WWTPs, in which the municipal wastewater which contains bacteria present in human digestive tract is the main source of bioaerosols, the studies also included coliform and psychrophilic bacteria. Such microorganisms are a good indicator of air contamination and were also studied by several other authors (Carducci et al. 2000; Korzeniewska et al. 2009; Michałkiewicz et al. 2011).

The air samples (50–100 dm^3^) were collected using the sedimentation, aspiration, and impaction methods using a MAS 100-Eco microbial air sampler (Merck). During the sampling of air for microbial studies, the microclimatic parameters such as temperature (°C) and relative humidity (%) as well as wind velocity (m/s) and direction were also analyzed. The measurements were carried out using an AZ8911 anemo-psychrometer (AZ Instrument Corp.). After collecting the air samples, all Petri dishes with the appropriate media were placed in incubators in order to ensure incubation of microorganisms. The type of medium, growth time, and the temperature was in accordance with the guidelines of Polish Standards. Mesophilic bacteria were grown using nutrient agar at 37 °C for 48 h, M+ and M− staphylococci were grown using Chapman medium at 37 °C for 48 h, *Actinobacteria* were grown using a Pochon medium at 26 °C for 120 h, *Pseudomonas fluorescens* was grown using a King B medium at 26 °C for 120 h and at 4 °C for 168 h with additional identification of colonies in UV rays, and microscopic fungi were grown on Waksman and Czapek-Dox media at 26 °C for 168 h. The coliform bacteria, which were not included in these standards, were grown on an Agar Endo medium at 37 °C for 48 h while psychrophilic bacteria were grown on nutrient agar at 20 ± 2 °C for 72 h. After the incubation period, the colonies were counted and the final result was expressed as the number of colony forming units calculated per a single cubic meter of air (CFU m^−3^). In the case of samples collected using the impaction method, the obtained results were corrected in accordance with Fellers conversion table. The obtained results were subjected to statistical analysis based on the Kruskal-Wallis test and analysis of Spearman’s rank correlation coefficients. All statistical analyses were carried out using the STATISTICA 10 PL software.

## Results and discussion

Table 2 presents the minimum (Min.), maximum (Max.), and median (Med.) abundance of the studied bacteria and microscopic fungi (CFU m^−3^) obtained in the research stations throughout the annum, whereas Table 3 presents the same measurements but obtained at the control stations, in the background studies. Due to the lack of a normality of abundance of bacteria and microscopic fungi, a non-parametric Kruskal-Wallis test was used to compare the microorganisms during the four seasons and in the annual cycle in the 11 studied WWTPs in order to investigate the differences in the average abundance level of these microorganisms. The value of *p* < 0.05 was assumed as statistically significant. Based on the comparison of results obtained during studies of bacteria and microscopic fungi at the research stations located in WWTPs (Table 2) with the results of background studies (Table 3), it can be established that the lowest microbial abundance and the lowest microbiological contamination of air was usually observed at the control stations (CFU m^−3^). The maximum abundance of selected bacteria, e.g., mesophilic bacteria, was lower at control stations by 7 (no. 11) to 2438 times (no. 1) compared to the WWTPs, while in the case of fungi grown on the Waksman medium, the abundance was lower by 2 (no. 8) to 121 times (no. 11). Even higher differences occurred in the case of coliform bacteria and *Pseudomonas fluorescens*. These values indicate that wastewater treatment plants are a significant source of air contamination.

**Table 2:** The minimum (Min.), maximum (Max.), and median (Med.) values of the number of bacteria and microscopic fungi tested (CFU m^−3^) in the treatment plants studied throughout the year

Microorganisms and determination		WWTP no. (research station on the WWTP area)										
		1	2	3	4	5	6	7	8	9	10	11
Mesophilic bacteria	Min.	50	20	80	80	0	80	10	40	10	80	30
	Max.	195,000	124,000	70,780	16,990	8340	16,500	16,500	3200	8040	89,120	3400
	Med.	320	200	590	790	1180	550	1300	360	160	670	184
Psychrophilic bacteria	Min.	80	40	80	1020	320	700	650	140	40	50	530
	Max.	225,000	157,800	84,230	15,100	29,260	19,000	38,000	7320	48,640	55,180	307,600
	Med.	1350	1250	2280	3615	4800	2400	3700	760	820	1180	2983
Coliform bacteria	Min.	0	0	0	0	0	0	0	0	0	0	0
	Max.	1150	57,800	87,530	3000	660	3000	3900	500	80	7320	200
	Med.	26	0	0	0	0	0	26	10	0	5	10
M− staphylococci	Min.	0	0	0	0	0	0	0	0	0	0	0
	Max.	470	120	320	160	80	160	240	170	60	930	200
	Med.	0	0	0	0	0	0	0	0	0	0	10
M+ staphylococci	Min.	0	0	0	0	0	0	0	0	0	0	0
	Max.	262	50	600	130	110	680	1400	110	150	40	160
	Med.	26	0	79	0	26	0	26	10	20	0	10
*Pseudomonas fluorescens* at 26 °C	Min.	0	0	0	0	0	0	0	0	0	0	0
	Max.	990	1570	3150	130	1210	80	80	20	120	460	80
	Med.	26	0	0	0	0	0	0	0	0	0	0
*Pseudomonas fluorescens* at 4 °C	Min.	ns	0	0	0	0	0	0	0	0	0	0
	Max.	ns	10	80	0	0	0	0	0	0	0	0
	Med.	ns	0	0	0	0	0	0	0	0	0	0
*Actinobacteria*	Min.	ns	ns	ns	ns	ns	ns	ns	0	0	0	0
	Max.	ns	ns	ns	ns	ns	ns	ns	380	6200	330	1600
	Med.	ns	ns	ns	ns	ns	ns	ns	80	75	10	40
Microscopic fungi: Waksman	Min.	160	40	160	160	390	80	650	500	40	40	530
	Max.	265,000	90,800	65,740	46,630	44,120	9700	34,000	6560	26,000	6040	1,148,530
	Med.	2670	1780	2125	4440	2830	1500	3900	1300	1120	740	3334
Microscopic fungi: Czapek-Dox	Min.	ns	40	0	0	390	240	1100	260	10	40	130
	Max.	ns	157,800	50,170	16,670	33,660	22,400	59,000	11,900	12,440	89,120	1,034,270
	Med.	ns	1250	1610	4325	2280	2000	6700	1010	740	840	2250

*ns*, not studied

**Table 3:** The minimum (Min.), maximum (Max.), and median (Med.) values of the counted bacteria and microscopic fungi (CFU m^−3^) at control positions throughout the year

Microorganisms and determination		WWTP no. (control station)										
		1	2	3	4	5	6	7	8	9	10	11
Mesophilic bacteria	Min.	0	40	79	0	52	40	80	60	20	40	33
	Max.	80	140	430	240	540	225	400	160	120	320	467
	Med.	78	40	160	54	205	60	160	90	60	190	150
Psychrophilic bacteria	Min.	0	60	200	650	300	400	550	40	60	240	333
	Max.	3500	820	1340	1260	780	1000	2400	860	1780	740	4400
	Med.	167	390	770	840	545	550	750	250	470	610	2684
Coliform bacteria	Min.	0	0	0	0	0	0	0	0	0	0	0
	Max.	0	0	0	79	0	0	0	0	0	0	60
	Med.	0	0	0	0	0	0	0	0	0	0	10
M− staphylococci	Min.	0	0	0	0	0	0	0	0	0	0	0
	Max.	0	0	26	0	0	0	0	0	10	20	60
	Med.	0	0	0	0	0	0	0	0	0	10	10
M+ staphylococci	Min.	0	0	0	0	0	0	0	0	0	0	0
	Max.	0	0	52	0	0	0	0	0	0	0	20
	Med.	0	0	26	0	0	0	0	0	0	0	0
*Pseudomonas fluorescens* at 26 °C	Min.	0	0	0	0	0	0	0	0	0	0	0
	Max.	52	0	0	0	0	0	0	0	0	0	0
	Med.	0	0	0	0	0	0	0	0	0	0	0
*Pseudomonas fluorescens* at 4 °C	Min.	ns	0	0	0	0	0	0	0	0	0	0
	Max.	ns	0	0	0	0	0	0	0	0	0	0
	Med.	ns	0	0	0	0	0	0	0	0	0	0
*Actinobacteria*	Min.	ns	ns	ns	ns	ns	ns	ns	30	0	0	0
	Max.	ns	ns	ns	ns	ns	ns	ns	50	3780	140	520
	Med.	ns	ns	ns	ns	ns	ns	ns	40	560	10	50
Microscopic fungi: Waksman	Min.	0	40	260	150	200	40	325	340	100	160	733
	Max.	13,600	1840	3790	3540	5380	1300	3200	3340	2020	840	9500
	Med.	315	370	1240	1355	1520	380	950	470	840	785	5233
Microscopic fungi: Czapek-Dox	Min.	ns	80	100	120	210	150	800	120	40	100	267
	Max.	ns	540	7540	2590	1610	1000	2400	3500	1440	710	3733
	Med.	ns	160	980	1410	1080	638	875	290	940	520	1750

*ns*, not studied

Occasional occurrence of slightly higher quantities of some microorganisms at control stations (e.g., psychrophilic bacteria or microscopic fungi) may be associated with the change of wind direction or accidental transfer of microorganisms from other areas. In the case of WWTPs, usually the microscopic fungi were present at highest concentrations (Max. 1,148,530 CFU m^−3^), followed by psychrophilic bacteria (Max. 307,600 CFU m^−3^) and mesophilic bacteria (Max. 195,000 CFU m^−3^). The coliform bacteria were also present on the area of each WWTP (Max. 57,800 CFU m^−3^), which is a good indicator of the presence of bioaerosol originating from wastewater or sludge. The staphylococci and *Pseudomonas fluorescens* were present at considerably lower levels. *Actinobacteria* were detected only in four WWTPs and their abundance was variable. Considering the maximum abundances of the studied bacteria and microscopic fungi in the annual cycle and the assessment of air in accordance with Polish Standards PN-89/Z-04111/02 and PN-89/Z-04111/03, it can be established that a periodic strong contamination of air with bacteria as well as contamination which is hazardous for the human environment due to the presence of microscopic fungi was confirmed on the area of each WWTP. Adamus-Białek et al. (2015) reported that the results of their studies indicate a high number of bacteria even at a relatively far distance from WWTPs, which allows these municipal objects to have a negative influence on the contamination of air in the surrounding areas, whereas M+ staphylococci as well as *Pseudomonas fluorescens* may be a notable component of the bioaerosol, which is hazardous for WWTP employees as well as other people in the surrounding areas.

On the basis of the Kruskal-Wallis test (statistical significance at *p* < 0.05), the comparison of median abundance values of mesophilic bacteria (*p* = 0.0000), psychrophilic bacteria (*p* = 0.0000), coliform bacteria (*p* = 0.0010), mannitol-negative (*p* = 0.0252) and mannitol-positive (*p* = 0.0000) staphylococci, *Pseudomonas fluorescens* cultivated at 26 °C (*p* = 0.0000), and *Actinobacteria* (*p* = 0.0309) during the annual cycle revealed statistically significant differences among the studied groups. A similar relation (*p* < 0.05) was also noted on the areas of studied WWTPs in each season (spring, summer, autumn, and winter), with the exception of coliform bacteria in summer (*p* = 0.0810). Due to the fact that *Pseudomonas fluorescens* cultivated at 4 °C was present only in three measurements in the area of two WWTPs, in this case, no detailed statistical analysis was carried out. The comparison of median abundance values of microscopic fungi in the annual cycle cultivated using the Czapek-Dox and Waksman media also revealed statistically significant differences among them (*p* = 0.0000). A similar relation was also noted in the case of fungi during specific seasons.

In order to present the changes of abundance of bacteria and microscopic fungi during specific seasons (spring, summer, autumn, winter) at the area of all studied wastewater treatment plants, the results of statistical calculations and Kruskal-Wallis test at appropriate research stations are presented in Table 4. The assumed level of statistical significance for the calculations was *p* < 0.05.

**Table 4:** Basic descriptive statistics and Kruskal-Wallis test results for the abundance of bacteria and microscopic fungi during the four seasons at the area of all studied wastewater treatment plants (no. 1–11)

Season	*n*	Average value ($\overline{x}$)	Standard deviation (SD)	Median value (Med.)	Minimum value (Min.)	Maximum value (Max.)	Bottom quartile Q_1_ (25%)	Upper quartile Q_3_ (75%)	Range (*R*)	Kruskal-Wallis test	
										*H*	*p*
Mesophilic bacteria (CFU m^−3^)											
SP	81	925	1470	280	33	7390	120	1100	7357	11.74	0.0083*
SU	89	3654	11,356	550	79	89,120	220	2000	89,041		
AU	98	5614	25,117	315	10	195,000	120	790	194,990		
WI	93	3334	9779	325	0	70,780	120	1940	70,780		
Psychrophilic bacteria (CFU m^−3^)											
SP	81	9263	42,444	1220	40	307,600	520	3867	307,560	27.38	0.0000*
SU	89	7638	13,654	3000	280	82,780	1460	7020	82,500		
AU	98	9073	30,649	1670	40	225,000	820	3700	224,960		
WI	93	5801	18,695	1100	40	157,800	600	3560	157,760		
Coliform bacteria (CFU m^−3^)											
SP	81	92	443	0	0	3900	0	26	3900	20.11	0.0002*
SU	89	117	572	0	0	4480	0	26	4480		
AU	98	1721	10,553	20	0	87,530	0	52	87,530		
WI	85	49	170	0	0	1080	0	10	1080		
M− staphylococci (CFU m^−3^)											
SP	81	17	33	0	0	180	0	20	180	0.81	0.8482
SU	89	24	47	0	0	240	0	26	240		
AU	98	31	110	0	0	930	0	26	930		
WI	93	20	42	0	0	240	0	20	240		
M+ staphylococci (CFU m^−3^)											
SP	81	29	65	10	0	390	0	26	390	2.77	0.4284
SU	89	20	30	0	0	130	0	26	130		
AU	98	43	96	10	0	600	0	40	600		
WI	93	69	178	10	0	1400	0	52	1400		
*Pseudomonas fluorescens* at 26 °C (CFU m^−3^)											
SP	81	20	48	0	0	314	0	26	314	1.26	0.7393
SU	89	17	54	0	0	460	0	10	460		
AU	98	77	379	0	0	3150	0	20	3150		
WI	93	51	160	0	0	990	0	13	990		
*Pseudomonas fluorescens* at 4 °C (CFU m^−3^)											
SP	74	1,1	9,2	0	0	80	0	0	80	n.a.	n.a.
SU	79	0,1	1,1	0	0	10	0	0	10		
AU	79	0,0	0,0	0	0	0	0	0	0		
WI	79	0,1	1,1	0	0	10	0	0	10		
*Actinobacteria* (CFU m^−3^)											
SP	32	113	123	70	0	580	35	160	580	63.83	0.0000*
SU	32	1006	1384	400	0	6200	130	1620	6200		
AU	32	21	34	0	0	160	0	35	160		
WI	32	42	85	5	0	380	0	45	380		
Microscopic fungi: Waksman (CFU m^−3^)											
SP	81	25,327	141,609	920	40	1,148,530	360	1800	1,148,490	85.66	0.0000*
SU	89	9179	16,173	4467	80	119,667	2310	7940	119,587		
AU	98	7926	28,273	2700	200	265,000	1700	5600	264,800		
WI	93	4911	21,087	800	40	188,700	390	1467	188,660		
Microscopic fungi: Czapek-Dox (CFU m^−3^)											
SP	73	35,655	158,170	1580	40	1,034,270	680	4780	1,034,230	88.73	0.0000*
SU	79	10,975	18,524	4420	440	89,120	2000	9830	88,680		
AU	79	5745	21,003	1560	560	157,600	960	3780	157,040		
WI	79	3794	18,139	533	0	157,800	160	1000	157,800		

*n*, number of measurements; *H*, value of Kruskal-Wallis test; *p*, test probability level; *n.a.*, not analyzed; *SP*, spring; *SU*, summer; *AU*, autumn; *WI*, winter; *statistically significant difference, *p* < 0.05

The highest median value for the majority of microorganisms (mesophilic bacteria, psychrophilic bacteria, *Actinobacteria*, fungi grown on Waksman and Czapek-Dox media) occurred in summer or autumn (coliform bacteria), whereas the lowest occurred in winter (psychrophilic bacteria, microscopic fungi grown on both media). In the case of some microorganisms (M− staphylococci, *Pseudomonas fluorescens* at 26 °C and 4 °C), the median value for all seasons was equal to 0. The comparison of median abundance of mesophilic bacteria (*p* = 0.0083), psychrophilic bacteria (*p* = 0.0000), coliform bacteria (*p* = 0.0002), and *Actinobacteria* (*p* = 0.0000) as well as fungi grown on the Waksman medium (*p* = 0.0000) and the Czapek-Dox medium (*p* = 0.0000) during four seasons indicated statistically significant differences among them (*p* < 0.05). On the other hand, the comparison of median abundance of M+ staphylococci (*p* = 0.4284), M− staphylococci (*p* = 0.8482) and *Pseudomonas fluorescens* grown at 26 °C (*p* = 0.7393) during four seasons based on the Kruskal-Wallis test did not reveal any statistically significant differences among them. After analysis of average values, it was established that their highest values do not always match the highest median values. In most cases, the highest average values occurred during autumn (mesophilic bacteria, coliform bacteria, M− staphylococci, *Pseudomonas fluorescens* at 26 °C) or spring (psychrophilic bacteria, *Pseudomonas fluorescens* at 4 °C, microscopic fungi: Waksman and Czapek-Dox medium) at slightly lower temperatures of the environment.

**Fig. 2:**
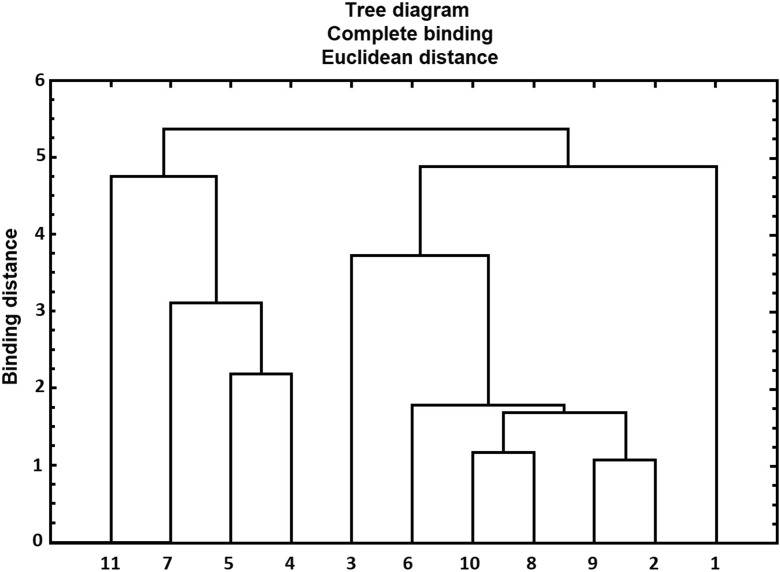
Dendrogram determined by the full binding method for 11 locations

Based on the median abundance value for bacteria and microscopic fungi (CFU m^−3^) from all stations in a given location during the annual cycle (Table 2), a cluster analysis and comparison of the studied WWTPs were conducted. Due to the fact that all types of studied microorganisms were not always present on the areas of the analyzed WWTPs, the comparison was conducted only in terms of 7 microbial groups which were present in all 11 studied locations. The comparison included mesophilic bacteria, psychrophilic bacteria, coliform bacteria, M− and M+ staphylococci, *Pseudomonas fluorescens* cultivated at 26 °C, and microscopic fungi cultivated using Waksman media. The dendrogram determined by full binding method for all (11) studied locations was presented in Fig. 2.

On the basis of calculations and Fig. 2, there were four clusters of WWTPs at a binding distance level equal to 4:

1. WWTP in location no. 11
2. WWTPs in locations no. 7, 5 and 4
3. WWTPs in locations no. 3, 6, 10, 8, 9 and 2
4. WWTP in location no. 1

In contrast, there were six WWTPs at a binding distance level equal to 2.5:

1. WWTP in location no. 11
2. WWTP in location no. 7
3. WWTPs in locations no. 5 and 4
4. WWTP in location no. 3
5. WWTPs in locations no. 6, 10, 8, 9 and 2
6. WWTP in location no. 1

In both analyzed cases, it is clearly visible that WWTPs in location nos. 11 and 1 differ from the rest. These are mechanical-biological treatment plants, which comprise additional composting stations, big open digesters and use a surface aeration system (no. 1) and posses a big, partially open sludge dehydration station (no. 11). In the case of WWTP no. 11, the presence of M− staphylococci was detected in more than half of the measurements, whereas in WWTP no. 1, more than half of the measurements indicated the presence of *Pseudomonas fluorescens* grown at 26 °C. WWTP nos. 6, 10, 8, 9, and 2 were the most similar in terms of abundance and presence of specific groups of microorganisms. Despite the fact that these objects are characterized by different capacity (no. 6–350 m^3^/day, no. 8–200,000 m^3^/day), their microbial similarity is high. Some of the objects were hermetized; biological treatment was conducted in Bardenpho-type bioreactors, modified Bardenpho systems, LANR II, and Lemna-type ponds; and fine bubble aeration systems (membrane and tube Magnum- and HAFI-type diffusers) were used for aeration. The similarity and formation of clusters of specific WWTPs were decided based on the type of studied microorganisms and their domination and abundance as well as any additional sources of air contamination (in this case, the presence of, e.g., composting stations), while no similarity in terms of capacity was observed for these objects.

During the collection of air samples, the microclimatic conditions at specific stations were investigated as well. All research stations were taken into account during the statistical analysis, including background studies. Table 5 presents the basic descriptive statistics (*n*, number of measurements; $\overline{x}$, average value; SD, standard deviation; Med., median value; Min., minimum value; Max., maximum value; Q_1_ bottom and Q_3_ upper quartile; *R*, range) regarding microclimatic parameters at specific study periods (seasons) and the annum. The air temperature was diverse and in most cases ranged from 10 to 20 °C (42.7% of all measurements). Air humidity was very variable and in most cases ranged between 50 and 60% (23.1% of all measurements), whereas the wind velocity in most cases reached up to 1 m/s (43.6% of all measurements).

**Table 5:** Descriptive statistics of microclimatic parameters

Study period	*n*	Average value ($\overline{x}$)	Standard deviation (SD)	Median value (Med.)	Minimum value (Min.)	Maximum value (Max.)	Bottom quartile Q_1_ (25%)	Upper quartile Q_3_ (75%)	Range (*R*)
Temperature (°C)									
Spring	94	16.0	5.3	16.9	3.0	25.3	12.7	20.1	22.3
Summer	102	23.6	5.9	21.7	15.4	39.3	19.4	27.0	23.9
Autumn	114	11.3	5.2	11.8	− 1.0	22.5	7.9	14.6	23.5
Winter	105	5.8	5.8	6.5	− 13.5	17.8	4.3	9.1	31.3
Total annum	415	14.0	8.6	13.6	− 13.5	39.3	7.8	19.8	52.8
Humidity (%)									
Spring	94	43.6	12.9	41.0	24.0	74.0	33.0	50.0	50.0
Summer	102	47.7	14.0	50.5	24.0	81.0	36.0	57.0	57.0
Autumn	114	69.9	13.7	70.5	37.0	99.8	59.2	79.0	62.8
Winter	105	64.1	12.9	62.0	42.0	94.0	55.0	73.0	52.0
Total annum	415	57.0	17.3	56.0	24.0	99.8	44.0	69.0	75.8
Wind velocity (m/s)									
Spring	82	1.2	0.9	0.8	0.0	3.5	0.5	1.5	3.5
Summer	90	1.3	0.7	1.3	0.0	3.5	0.8	1.6	3.5
Autumn	102	1.3	1.0	1.2	0.0	3.4	0.5	2.0	3.4
Winter	93	1.8	1.2	1.4	0.1	5.3	0.9	2.6	5.2
Total annum	367	1.4	1.0	1.2	0.0	5.3	0.6	2.0	5.3

In order to check the relations occurring between the abundance of studied groups of bacteria as well as microscopic fungi and the microclimatic parameters (air temperature and humidity), calculations of Spearman’s rank correlation coefficients for specific WWPTs (locations) were carried out. Positive correlations indicate that the higher the analyzed parameters, the higher the abundance of specific microorganisms, whereas negative correlations indicate that the abundance of microorganisms is lower with higher parameters. Interpretation of results was based on the classification of Spearman’s rank correlation coefficients according to Guilford (Świechowski et al. 2015): |*r*| = 0 variables are not correlated; 0.0 < |*r*| ≤ 0.1 dim correlation; 0.1 < |*r*| ≤ 0.3 weak correlation; 0.3 < |*r*| ≤ 0.5 average correlation; 0.5 < |*r*| ≤ 0.7 strong correlation; 0.7 < |*r*| ≤ 0.9 very strong correlation; 0.9 < |*r*| < 1.0 almost complete correlation; and |*r*| = 1 complete correlation.

The highest number of correlations between the abundance of the studied microorganisms and air temperature was observed in the case of microscopic fungi. In this case, all correlation coefficients were significant at *p* < 0.05. For fungi which were grown using the Waksman medium, average and high positive correlations (coefficients ranging from 0.409 to 0.700) at the area of 7 WWTPs and a negative correlation (− 0.559) for a single WWTP were observed. In general, a weak positive correlation was observed (0.352) for all the WWTPs. In the case of cultivation on the Czapek-Dox medium, only positive correlations (from weak to very strong, ranging from 0.280 to 0.857) and an average positive correlation (0.562) was established in general. Among the studied groups of bacteria, the most significant correlations between the bacterial abundance and air temperature occurred at the smallest WWTP. In four WWTPs, a positive correlation among psychrophilic bacteria was observed, for which the Spearman’s range of correlation coefficient ranged from 0.280 to 0.712 and an average positive correlation (0.352) was observed for all WWTPs in general. Additionally, in the case of four WWTPs, significant correlations for M− staphylococci were observed as well. Three were positive, average, and high (from 0.433 to 0.588) and a single was negative and weak (− 0.277). A lower number of significant correlations (for two or three WWTPs) was observed in the case of mesophilic bacteria (− 0.386, 0.293 and 0.345), coliform bacteria (0.433 and 0.642), *Actinobacteria* (0.425 and 0.846) or M+ staphylococci (− 0.467 and − 0.650), and air temperature. A weak negative correlation (− 0.378) between the abundance of *Pseudomonas fluorescens* and air temperature was observed only in a single WWTP.

By analyzing the significant correlations between the abundance of the studied microorganisms and air humidity, it can be established that the highest numbers occurred in the case of microscopic fungi grown using the Waksman medium. This included average and high correlations noted for five WWTPs, for which the Spearman’s range of correlation coefficients ranged from 0.417 to 0.601, and negative correlations for two WWTPs (− 0.436 and − 0.651). In the case of cultivation using the Czapek-Dox medium, only a single significant negative correlation was observed (− 0.693). Among bacteria, the highest number of significant correlations between the microbial abundance and air humidity occurred only in three WWTPs and concerned coliform bacteria (− 0.488, 0.393, and 0.468) as well as M− staphylococci (− 0.586, − 0.488, and 0.414). In contrast, there was no significant correlation between the M+ staphylococci and air humidity in any of the WWTPs. Between the remaining bacterial groups (mesophilic and psychrophilic bacteria, *P. fluorescens*, *Actinobacteria*) and air humidity in most cases, there were positive and/or negative weak, average, or strong correlation in the case of a single WWTP (Spearman’s range of correlation coefficients ranged from − 0.609 to 0.492). The studies of other authors indicate that correlations between the microorganisms and climatic parameters are very diverse.

During their studies regarding bacterial aerosols in Gliwice (Upper Silesia, Poland), Brągoszewska et al. (2017) established that the main meteorological factors with major roles to play in bioaerosol concentration and their transport are air temperature, relative humidity, wind velocity, UV radiation, and seasonal variations of microbial abundance. Statistically, temperature and UV radiation were the most important meteorological factors in the viability of airborne bacteria. Wind velocity was found to have no statistically significant correlation with bacterial aerosol concentrations. Brągoszewska and Pastuszka (2018) reported that the average concentration of bacteria in the air in Gliwice (Poland) differed significantly between each season. In this case, positive correlations between the air temperature and bacterial bioaerosol were established in fall and winter (Spearman’s range of correlation coefficients was at 0.499 and 0.762, respectively) and negative correlations were observed in spring and summer (− 0.284 and − 0.687 for *p* < 0.05). A significantly higher positive correlation between the bacterial abundance and humidity (0.476) was observed only in winter. Partially similar correlations were noted in my studies in the case of selected groups of bacteria and air temperature, whereas no similar correlations were observed between bacterial abundance and air humidity.

Niazi et al. (2015) report that the highest emission of bacteria to air was observed during summer, whereas the emission of fungi was associated with mechanical treatment of wastewater. Overall, a significant relationship was observed between meteorological parameters and the concentration of bacterial and fungal aerosols. The authors found a significant correlation between the concentration of detected bacteria and temperature, UV index, and wind speed. Based on these results, temperature seems to have the highest correlation with bacterila concentration (*R* = 0.613, *p* < 0.001, *n* = 120). The studies of Kristanto and Rosana (2017) confirm that there is a significant correlation between air temperature and air humidity as well as wind speed and bacterial concentration (*p* = 0.012; *p* = 0.001; *p* = 0.015, respectively) and as for fungal concentration, there is only a significant correlation with air humidity (*p* = 0.000). Air humidity has the highest correlation with both the concentration of bacteria and fungi indicating that air humidity can influence bioaerosol concentration (the higher the humidity, the higher the concentration of bioaerosol). Mouli et al. (2005) also discussed the influence of meteorological factors (temperature, relative humidity, and wind velocity) on the occurrence of bacteria in Tirupati (India). Among these factors, wind speed had the highest influence on bacterial concentration with a regression coefficient (*β*) ranging from 0.225 to 2.092, followed by temperature, for which the regression coefficient (*β*) ranged from 0.740 to − 0.745. Wind speed showed a very good positive correlation at each location signifying that the bacteria concentration will increase with increasing wind velocity. In contrast, relative humidity (RH) showed a relatively low influence on airborne bacteria. The results of my studies did not confirm the statements of Mouli et al. (2005) regarding the existence of a significant correlation between wind speed and microbial abundance.

During the study of bioaerosols on aircraft boards, Stobnicka et al. (2016) observed relatively low shifts of temperature (11.1–15.7 °C) and humidity (20–45%). Based on the results of Spearman’s correlations, the authors established that the air temperature had a significant influence on the concentration of bacteria in the air (*p* < 0.05), whereas it had no impact on the concentration of fungi (*p* > 0.05). The relative humidity did not determine the concentrations of bioaerosols in a significant manner (*p* > 0.05). According to Shravanthi et al. (2016), the number and composition of the air microflora in the premises of sewage treatment plants depend on the area of sewage exposed to atmospheric factors, the type and degree of sewage contamination, means of sewage management, atmospheric and climatic conditions, mixing and transportation of sewage, and gathering and lengthy storage of unstable biomass as well as elevated temperature are conducive to bioaerosols formation. While analyzing the levels of microbiological load in the air of a particular WWTP area (Visakhapatnam, India) depending on climatic conditions, a greater number of microbes was usually observed in the months of lower temperatures (winter) and stronger winds. On the contrary, Carducci et al. (2000) did not find any significant relationship between meteorological parameters and the number of bacterial colonies. A different set of data was reported by Korzeniewska et al. (2009), concluding that the majority of the analyzed groups of microorganisms was strongly correlated with the air temperature (with the exception of staphylococci and enterococci) or weakly correlated with air humidity (with the exception of fungi, yeast, psychrophilic and hemolytic bacteria). Partially different relations were observed in the framework of this study. In most cases, the temperature had a significant influence on the abundance of fungi (the higher the temperature, the higher the concentration of fungi), whereas in the case of bacteria, both negative and positive correlations were observed in some WWTPs (values ranging from − 0.650 to 0.846; *p* < 0.05). In several WWTPs, the relative humidity also had a significant positive influence on the concentration of fungi grown using the Waksman medium (from 0.417 to 0.601), whereas in the case of bacteria, the correlations were notably more rare and more diverse (both positive and negative). According to Han et al. (2018), the capacity of WWTP, distance from the emission source, the type and performance of devices used to separate the sludge, quality of wastewater, and possible hermetization of treatment stages had an influence on the emission of bioaerosols. The highest concentrations of bacteria were observed during summer at high temperature (above 28 °C) and humidity (55–85%). Therefore, it was concluded that humidity is positively correlated with microbial abundance. The studies of Fernando and Fedorak (2005) indicate that hermetization of objects and fine bubble aeration notably decrease the emission of microorganisms to air, whereas an intense, turbulent flow of wastewater and lack of ventilation increase the emission of bioaerosols. All research results obtained after the modernization of WWTP were statistically lower (*p* < 0.05). The authors concluded that the decrease of bioaerosol emission will positively affect the health of employees, which are exposed to pathogens.

The studies of Sabariego et al. (2000) were focused on the influence of meteorological factors on the changes of abundance of spores of different genera of airborne fungi in Granda (southern Spain). The results showed that the genera *Alternaria* and *Cladosporium* were positively correlated (*p* ≤ 0.01) with the temperature and sunlight. In contrast, the genera *Ustilago* showed negative correlation coefficients with temperature, sunlight, and wind speed and positive coefficients with relative air humidity. In my studies, it was established that the abundance of fungi was usually the highest in all wastewater treatment plants and in most cases, it was positively correlated with both temperature and air humidity.

## Conclusions

Studies of bioaerosol emission in 11 WWTPs indicated that a diverse abundance of bacteria and microscopic fungi emitted to the air environment can be observed at the subsequent wastewater treatment stages. In general, the microscopic fungi occurred at highest concentration, followed by psychrophilic and mesophilic bacteria, the abundance of which was very high at times. The maximum concentrations of microscopic fungi on the WWTPs ranged from 6040 to 1,148,530 CFU m^−3^ using the Waksman medium and from 11,900 to 1,034,270 CFU m^−3^ using the Czapek-Dox medium. The maximum concentration of bacteria varied for specific groups and ranged from 7320 to 307,600 CFU m^−3^ for psychrophilic bacteria, from 3200 to 195,000 CFU m^−3^ for mesophilic bacteria, and from 80 to 87,530 CFU m^−3^ in the case of coliform bacteria. The median values for fungi were more similar and oscillated between 740 and 6700 CFU m^−3^, whereas in the case of bacteria, the values were considerably lower and reached from 760 to 4800 CFU m^−3^ in the case of psychrophilic bacteria, from 160 to 1300 CFU m^−3^ in the case of mesophilic bacteria, and from 0 to 26 CFU m^−3^ in the case of coliform bacteria. The obtained results indicate that a periodic, strong air contamination occurs at the areas of wastewater treatment plants. In contrast, in the case of control stations, the concentrations of all microbial groups were multiple times lower compared to the areas of WWTPs. By analyzing the variable abundances of the studied microorganisms at specific seasons, it was established that the highest median value for the majority of microorganisms occurred in summer or autumn, while the lowest occurred in winter.

Based on the Kruskal-Wallis test (statistical significance at *p* < 0.05), the comparison of median abundance values for bacteria and microscopic fungi in an annual cycle revealed statistically significant differences between them. The median abundance values for bacteria and microscopic fungi were used to conduct a cluster analysis and compared the studied WWTPs. The calculations and the dendrogram indicate that at a binding distance level equal to 4, there were four WWTP clusters, whereas at the level of 2.5, there were six clusters. In both cases, two WWTPs were notably distinct from other objects, both of which contained an additional sludge composting site on their area.

The similarity of WWTPs was decided based on the dominance and abundance of microorganisms as well as additional contamination sources, whereas no clear similarities in terms of capacity were noted. Based on the calculation of Spearman’s range of correlation coefficients, it was established that the most significant correlations between the microbial abundance and temperature as well as humidity occurred in the case of microscopic fungi, whereas the correlations between bacteria and microclimatic parameters were less abundant and less diverse.

The obtained results allow to conclude that the studied wastewater treatment plants are a significant source of emissions of bacterial and fungal aerosols despite proper exploitation and correctly conducted treatment processes.

## Electronic supplementary material

ESM 1 (DOCX 23 kb)

## Abbreviations

AU: Autumn
CDT: Closed digester tanks
CFU m^−3^: Colony forming units
EC: Electrocoagulation process
ECP: Electrochemical peroxidation process
EF: Electro-Fenton process
*H*: Value of Kruskal-Wallis test
KO: Composting plant
L: Hydrobotanic Lemna-type treatment plant
M− staphylococci: Mannitol-negative staphylococci (*Staphylococcus*)
M+ staphylococci: Mannitol-positive staphylococci (*Staphylococcus*)
Max.: Maximum value
MB: Mechanical-biological Wastewater Treatment Plants
Med.: Median value
Min.: Minimum value
*n*: Number of measurements
n.a.: Not analyzed
ns: Not studied
ODT: Open digester tanks
*p*: Probability value for statistical significance testing
PE: Population equivalent
Q_1_: Bottom quartile
Q_3_: Upper quartile
*r*: Spearman’s rank correlation coefficients
*R*: Range
RH: Relative humidity
SD: Standard deviation
SP: Spring
SU: Summer
UV: UV radiation
WI: Winter
WWTPs: Wastewater treatment plants
*x̅*: Average value
*β*: Regression coefficient
