# Supplementary material for: Comparison of wastewater treatment plants based on the emissions of microbiological contaminants
Source: Environ Monit Assess. 2018 Oct 18;190(11):640. doi: 10.1007/s10661-018-7035-2 (PMC6208977; doi:10.1007/s10661-018-7035-2)
Supplement: Supplementary file 1 — (DOCX 23 kb) [file 10661_2018_7035_MOESM1_ESM.docx]

1. Adamus-Białek. DOI: 10.2429/proc.2015.9(2)047. <http://tchie.uni.opole.pl/PECO15_2/EN/AdamusBialekWawszczak_PECO15_2.pdf>

2. Ahmadzadeh. DOI: 10.1016/j.psep.2017.04.026. <https://www.sciencedirect.com/science/article/pii/S095758201730143X>, <https://kundoc.com/pdf-removal-of-ciprofloxacin-from-hospital-wastewater-using-electrocoagulation-techn.html>

3. Ahmadzadeh. DOI: 10.5004/dwt.2017.21492. <http://www.deswater.com/vol.php?vol=92&oth=92%7C0%7COctober%7C2017>

4. Ahmadzadeh. DOI: 10.1007/s12665-017-7203-7. <https://link.springer.com/article/10.1007/s12665-017-7203-7>

5. Ahmadzadeh. DOI: 10.1007/s10661-018-6697-0. <https://link.springer.com/article/10.1007%2Fs10661-018-6697-0>

6. Ahmadzadeh. DOI: 10.1016/j.molliq.2018.01.080. <https://www.sciencedirect.com/science/article/pii/S0167732217354387>

7. Bauer. DOI:10.1016/S0169-8095(02)00084-4. <https://www.sciencedirect.com/science/article/pii/S0169809502000844>

8. Blanchard. DOI:10.1126/science.170.3958.626. <http://science.sciencemag.org/content/170/3958/626>

9. Brągoszewska. DOI: 10.1007/s11869-018-0579-z. <https://link.springer.com/content/pdf/10.1007%2Fs11869-018-0579-z.pdf>

10. Brągoszewska. DOI:10.3390/atmos8120239. <https://www.mdpi.com/2073-4433/8/12/239/htm>

11. Brągoszewska. DOI:10.1007/s10453-018-9510-1. <https://link.springer.com/content/pdf/10.1007%2Fs10453-018-9510-1.pdf>

12. Carducci. DOI[:10.1016/S0043-1354(99)00264-X](https://doi.org/10.1016/S0043-1354(99)00264-X). <https://www.sciencedirect.com/science/article/pii/S004313549900264X>

13. Dungan. DOI:[10.1016/j.envint.2011.12.004](https://doi.org/10.1016/j.envint.2011.12.004). <https://www.sciencedirect.com/science/article/pii/S0160412011002844?via%3Dihub>

14. Fernando. DOI:10.1016/j.watres.2005.08.010. <https://www.sciencedirect.com/science/article/pii/S0043135405004641?via%3Dihub>

15. Fouladgar. DOI: 10.1016/j.apsusc.2016.04.026. <https://www.sciencedirect.com/science/article/pii/S016943321630767X>

16. Fuzzi. DOI:10.5194/acp-6-2017-2006. <https://www.atmos-chem-phys.net/6/2017/2006/acp-6-2017-2006.pdf>

17. Górny. <http://www.aaem.pl/Bacterial-and-fungal-aerosols-in-indoor-environment-in-Central-and-Eastern-European,72765,0,2.html>

18. Gregová. <http://www.uvlf.sk/document/folia-veterinaria-volume-52-issue-2.pdf>

19. Han. [DOI:10.1016/j.scitotenv.2017.11.071](https://doi.org/10.1016/j.scitotenv.2017.11.071). <https://www.researchgate.net/publication/321082461_Bacterial_population_and_chemicals_in_bioaerosols_from_indoor_environment_Sludge_dewatering_houses_in_nine_municipal_wastewater_treatment_plants>

20. Kassim. DOI: 10.1088/1757-899X/17/1/012010. <http://iopscience.iop.org/article/10.1088/1757-899X/17/1/012010/pdf>

21. Korzeniewska. DOI:[10.1016/j.watres.2009.03.050](https://doi.org/10.1016/j.watres.2009.03.050). <https://www.sciencedirect.com/science/article/pii/S0043135409001870?via%3Dihub>

22. Kristanto. DOI: 10.1051/matecconf/201713808004. <https://www.matec-conferences.org/articles/matecconf/pdf/2017/52/matecconf_eacef2017_08004.pdf>

23. Li. <http://www.pjoes.com/Issue-5-2012,3846>

24. Maki. DOI:10.5194/acp-17-11877-2017. <https://www.atmos-chem-phys.net/17/11877/2017/acp-17-11877-2017.pdf>

25. Mandal. DOI:[10.2174/1875040001104010083](http://dx.doi.org/10.2174/1875040001104010083). <https://benthamopen.com/contents/pdf/TOEBMJ/TOEBMJ-4-83.pdf>

26. Mentese. DOI:10.1177/1420326X14562454. <http://journals.sagepub.com/doi/10.1177/1420326X14562454>

27. Michałkiewicz. <http://www.pjoes.com/Issue-5-2011,3840>

28. Mouli. <http://citeseerx.ist.psu.edu/viewdoc/download;jsessionid=085ED7A6BE4AC668D586B9A78FDDFE55?doi=10.1.1.581.2650&rep=rep1&type=pdf>

29. Niazi. DOI:10.1007/s11356-015-4793-z. <https://www.researchgate.net/publication/278042997_Assessment_of_bioaerosol_contamination_bacteria_and_fungi_in_the_largest_urban_wastewater_treatment_plant_in_the_Middle_East>

30. O’Connor. [DOI:10.1016/j.wasman.2015.04.015](https://doi.org/10.1016/j.wasman.2015.04.015). <https://www.sciencedirect.com/science/article/pii/S0956053X15002871?via%3Dihub>

31. Pardakhty. DOI: 10.1016/j.molliq.2016.01.010. <https://www.infona.pl/resource/bwmeta1.element.elsevier-ff11bd7e-afee-389f-a733-c4a55cbb2872>

32. Pastuszka. DOI:10.5277/epe130110. <http://epe.pwr.wroc.pl/2013/1-2013/Pastuszka_1-2013.pdf>

33. PN-89/Z-04111/02. (1989). <http://sklep.pkn.pl/pn-z-04111-02-1989p.html>

34. PN-89/Z-04111/03. (1989). <http://sklep.pkn.pl/pn-z-04111-03-1989p.html>

35. Pringle. [DOI:10.1371/journal.ppat.1003371](https://doi.org/10.1371/journal.ppat.1003371). <https://journals.plos.org/plospathogens/article/file?id=10.1371/journal.ppat.1003371&type=printable>

36. Roodbari. DOI:10.5277/epe130409. <http://epe.pwr.wroc.pl/2013/4-2013/Roodbari_4-2013.pdf>

37. Sabariego. DOI: 10.1007/s004840050131. <https://www.researchgate.net/publication/12440396_The_effect_of_meteorological_factors_on_the_daily_variation_of_airborne_fungal_spores_in_Granada_southern_Spain>

38. Sánchez-Monedero. DOI:[10.1016/j.watres.2008.06.028](https://doi.org/10.1016/j.watres.2008.06.028). <https://www.sciencedirect.com/science/article/pii/S0043135408002716?via%3Dihub>

### 39. Shravanthi. <https://www.worldwidejournals.com/international-journal-of-scientific-research-(IJSR)//articles.php?val=NzcyNg==&b1=157&k=40>

40. [Soltani](https://www.sciencedirect.com/science/article/pii/S0167732215313271#!). DOI: 10.1016/j.molliq.2016.03.014. <https://www.sciencedirect.com/science/article/pii/S0167732215313271>

41. Stobnicka. <http://ros.edu.pl/images/roczniki/2016/No2/37_ROS_N2_V18_R2016.pdf>.

42. Świechowski. <http://yadda.icm.edu.pl/yadda/element/bwmeta1.element.psjd-6306d68c-6859-4321-8f28-e64a6225a9e3>.

43. Thorn. [DOI:10.1093/annhyg/mef068](https://doi.org/10.1093/annhyg/mef068). <https://academic.oup.com/annweh/article/46/6/549/154102>

44. Tomasi. [DOI:10.1002/9783527336449.ch1](https://doi.org/10.1002/9783527336449.ch1). <https://application.wiley-vch.de/books/sample/3527336451_c01.pdf>

45. Uhrbrand. DOI:10.1007/s12560-011-9068-3. <https://link.springer.com/article/10.1007/s12560-011-9068-3>

46. Wéry. DOI:[10.3389/fcimb.2014.00042](https://dx.doi.org/10.3389%2Ffcimb.2014.00042). <https://www.frontiersin.org/articles/10.3389/fcimb.2014.00042/full>

47. Yoosefian. DOI: 10.1016/j.molliq.2016.11.093. <https://www.sciencedirect.com/science/article/pii/S0167732216328215>
